# Global Burden and Temporal Trends of Brain and Central Nervous System Tumors in Children and Adolescents: A Systematic Analysis for the Global Burden of Disease Study 2021

**DOI:** 10.1002/cai2.70079

**Published:** 2026-08-23

**Authors:** Zhaoyang Feng, Yu Su, Lin Yang, Kaiwen Deng, Jianmin Wang, Jiao Zhang, Fei Liu, Dongyang Wang, Yuyan Liang, Wei Wang, Xiaoguang Qiu, Tao Jiang, Yu Tian, Hailong Liu

**Affiliations:** ^1^ Department of Neurosurgery Beijing Tiantan Hospital, Capital Medical University Beijing China; ^2^ Department of Radiotherapy Beijing Tiantan Hospital, Capital Medical University Beijing China; ^3^ School of Public Health, Capital Medical University Beijing China; ^4^ Department of Hospital‐Acquired Infection Control Beijing Jishuitan Hospital Beijing China; ^5^ Clinical Oncology School of Fujian Medical University Fujian Cancer Hospital Fuzhou China; ^6^ Fujian Key Laboratory of Advanced Technology for Cancer Screening and Early Diagnosis, Clinical Oncology School of Fujian Medical University Fujian Cancer Hospital Fuzhou China; ^7^ Cancer and Hematology Center Texas Children's Hospital Houston Texas USA; ^8^ Department of Pediatrics‐Hematology/Oncology and Neurosurgery Baylor College of Medicine Houston Texas USA; ^9^ The Arthur and Sonia Labatt Brain Tumor Research Center The Hospital for Sick Children Toronto Canada; ^10^ Laboratory of Tumor Immunology, Beijing Pediatric Research Institute, Beijing Children's Hospital, Capital Medical University National Center for Children's Health Beijing China; ^11^ China National Clinical Research Center for Neurological Diseases, Beijing Tiantan Hospital, Capital Medical University Beijing China; ^12^ Beijing Neurosurgical Institute Beijing China; ^13^ Division of Cancer Epidemiology German Cancer Research Center (DKFZ) Heidelberg Germany; ^14^ Chinese Institute for Medical Research Beijing China

**Keywords:** age‐period‐cohort, brain tumors, child, global burden of disease, incidence, mortality

## Abstract

**Background:**

Central nervous system (CNS) tumors pose major global health challenges. Previous studies often assessed all‐age populations without focusing on children and adolescents. We analyzed the burden and temporal trends of CNS tumors among individuals aged < 20 years from 1990 to 2021, with projections to 2036.

**Methods:**

Data were obtained from the Global Burden of Disease Study 2021, including annual incidence, mortality, and disability‐adjusted life years (DALYs) for brain and CNS tumors. Regions were classified by Socio‐demographic Index (SDI) to examine associations between burden and development. Temporal trends were analyzed using the age‐period‐cohort (APC) model and future projections via the Bayesian APC model.

**Results:**

In 2021, an estimated 40,534.53 (95% uncertainty interval [UI]: 33,345.94–49,609.04) new cases of brain and CNS tumors occurred worldwide. The global age‐standardized incidence rate (ASIR) was 1.54 (95% UI: 1.27–1.88), and the death rate (ASDR) was 0.76 (0.61–0.93) per 100,000. High‐SDI regions showed the highest ASIR and ASDR, while lower‐SDI regions faced growing burdens. Mortality and DALYs improved most in Greenland, Serbia, and China. The APC model showed a mild global decline in incidence (*p* = 0.099) and significant mortality reduction (−1.16% annually, *p* < 0.01).

**Conclusions:**

Global CNS tumor burden declined but remains uneven. Enhanced prevention and healthcare access are needed, especially in low‐SDI regions.

## Graphical abstract

This study analyzed the global burden of central nervous system (CNS) tumors in individuals under 20 years from 1990 to 2021, revealing notable regional disparities. While overall incidence and mortality have gradually declined, low‐ and middle‐SDI regions continue to experience increasing burdens. Targeted interventions and international cooperation are crucial to reduce inequalities and improve outcomes for young patients worldwide.

**Abbreviations**
APC: age‐period‐cohort
ASIR: age‐standardized incidence rate
ASDR: age‐standardized death rate
ASDALYR: age‐standardized disability‐adjusted life year rate
BAPC: Bayesian age‐period‐cohort
CI: confidence interval
CNS: central nervous system
DALY: disability‐adjusted life year
GBD: global burden of disease
ICD: International Classification of Diseases
SDI: socio‐demographic index
UI: uncertainty interval
WHO: World Health Organization
RRs: relative risks

## 1 Introduction

Brain and central nervous system (CNS) tumors represent a leading cause of cancer‐related morbidity and mortality in children and adolescents globally, causing substantial clinical and socioeconomic consequences [1, 2]. Epidemiological analyses consistently demonstrate that these malignancies are the most common solid tumors in this population and rank among the top causes of childhood cancer deaths [3, 4, 5]. Additionally, a number of recent studies have highlighted the limitations of current therapeutic approaches, indicating the continuing difficulty and heavy burden of treatment for brain tumors [6, 7, 8, 9, 10]. The burden exhibits critical geographic disparities, with mortality rates in low‐resource settings substantially exceeding those in high‐income regions due to systemic inequities in healthcare access, diagnostic delays, and treatment limitations. This survival gap reflects broader health system challenges and contributes to catastrophic financial burdens for vulnerable families, particularly in regions with fragmented referral systems and limited neuro‐oncology capacity [11, 12].

Existing global burden assessments have primarily centered on either aggregate cancer statistics across all ages or broad pediatric cancer categories [13, 14, 15, 16]. Major initiatives such as international cancer registry networks and comprehensive disease burden studies provide invaluable population‐level perspectives on cancer epidemiology. However, parameters in these studies are often not analyzed in detail in pediatric populations or CNS tumors, including longitudinal trends across developmental stages (0–19 years). Furthermore, studies from well‐resourced healthcare systems offer critical benchmarks for survival outcomes; however, their frameworks rarely capture the complex interplay between socioeconomic determinants and clinical outcomes that characterize disparate regions. This may result in noteworthy knowledge gaps regarding the evolving disease burden across critical developmental windows and its intersection with health system disparities.

Using data from the Global Burden of Disease (GBD) 2021 study [17], our study aims to provide a systematic assessment of the global burden of CNS tumors for children and adolescents from 1990 to 2036. We analyzed the burden of incidence, mortality, and disability‐adjusted life years (DALYs) across various regions and countries from 1990 to 2021, including age‐standardization for further analysis. In addition, we utilized the Age‐Period‐Cohort (APC) model to analyze the temporal trends in the incidence and mortality rates of CNS tumors over the past 30 years (1992–2021), which provides insights into disease patterns across different age, period, and cohort effects. Finally, the Bayesian APC (BAPC) model was used to forecast future burden of CNS tumors. Overall, this comprehensive and in‐depth exploration elucidates trends in the global and regional distribution of childhood brain tumor burden in incidence, mortality, and epidemiology. This information could facilitate the formulation of health policies and the development of precision treatment strategies.

## 2 Materials and Methods

### 2.1 Data Collection

The GBD Study 2021 [17], which was produced by the Institute for Health Metrics and Evaluation, served as the primary data source for this study. This data set offers a comprehensive and up‐to‐date epidemiological insight into the burden associated with 371 diseases and injuries across 204 countries and territories. Data on brain and CNS tumors from 1990 to 2021 period were obtained through the Global Health Data Exchange (GHDx) query tool (https://vizhub.healthdata.org/gbd-results/) [17], including annual statistics on incidence, mortality, and DALYs. The corresponding population data (1992–2021) was used for APC analysis. In addition, our study referred to publicly available data, and no additional ethical consent was required.

The study population included children and adolescents aged 0–19 years, which differs from the traditional WHO definition of children as individuals younger than 18 years. Data associated with brain and CNS tumors were also extracted from the GBD 2021 database, all estimates and the 95% uncertainty intervals (UIs) for the incidence, mortality, and DALYs are generated from the 2.5th and 97.5th percentile values of 500 draws [17]. Countries with fewer or no data sources generally have a wider range of 95% UIs, suggesting greater inaccuracy in disease estimates. This analysis used the Socio‐demographic Index (SDI) for each country [18], an indicator estimated as a composite of income per capita, average years of schooling, and fertility rate in females under 25 years old. The SDI is scaled from 0 to 1, with higher values indicating higher socioeconomic levels. All countries were categorized into one of five SDI quintiles based on 2021 SDI values (Table S10).

All maps and regional visualizations presented in this study were constructed solely based on the territorial classifications and data structure provided by the GBD 2021 database. The separate listing or graphical representation of certain regions in accordance with the GBD 2021 database scheme does not imply any political position or endorsement of any territorial claims. These representations reflect only the statistical data organization as maintained by the Institute for Health Metrics and Evaluation.

For all age‐standardization, the standard population data was extracted from the World Health Organization's new World Standard Population, which was constructed from an average world population age‐structure for the period 2000–2025 [19]. Given that our analysis was restricted to individuals aged 0–19 years, only the corresponding age‐specific weights for this population were applied. These weights were proportionally rescaled to sum 1 within the 0–19 age range to ensure appropriate standardization for a pediatric‐only population [20]. Age‐standardized rates were calculated by direct method, which assumes that the rates are distributed as the weighted sum of independent Poisson random variables. According to the International Classification of Diseases, 10th revision (ICD‐10) codes, brain and CNS tumors were coded C70–C72.9 and C75.1–C75.3.

### 2.2 Age‐Period‐Cohort (APC) Model Analysis

To analyze temporal trends, the APC model framework was applied, which is recognized as an advanced research methodology that extends beyond traditional analyses in health and socio‐economic development studies. This model decomposes trends into age effects (natural history of the disease across life stages), period effects (calendar time changes), and cohort effects (shared experiences among individuals born in the same time period) [21, 22, 23]. The APC model estimates the net drift (overall annual percentage change) and local drift (age‐specific annual percentage change), as well as period and cohort relative risks (RRs) with corresponding 95% confidence intervals (CIs).

Given the inherent identifiability issue (exact linear dependency among age, period, and cohort), we adopted a conventional constraint‐based parameterization implemented in the APC Web Tool. In this approach, estimable functions (including net drift, local drift, and curvature components) are derived, while non‐identifiable linear components are incorporated into the drift term. The reference period and cohort were set to the median categories and do not affect the interpretation of estimable functions.

In APC analysis, age and period intervals are typically specified with equal widths, and thus 5‐year age groups were paired with 5‐year periods. In addition, this grouping is consistent with commonly used pediatric age classifications, reflecting key stages of physiological development and differences in clinical presentation and management across childhood and adolescence. Accordingly, we included data on brain and CNS tumors and the corresponding population from 1992 to 2021 at both the global level and across five SDI quintiles. The population aged 0–19 years was categorized into four groups (0–4, 5–9, 10–14, and 15–19 years). The study period was divided into six consecutive 5‐year intervals (1992–1996, 1997–2001, 2002–2006, 2007–2011, 2012–2016, and 2017–2021), yielding nine overlapping 10‐year birth cohorts from 1972 to 1981 to 2012–2021. The model estimated both overall temporal trends and age‐specific trends in incidence and mortality. Net drift represents the overall annual percentage change (% per year), whereas local drift reflects age‐specific annual percentage changes. The statistical significance of temporal trends was assessed using the Wald chi‐squared test.

### 2.3 Projection

For projection, we employed a BAPC model using integrated nested Laplace approximations. The BAPC framework assumes that age, period, and cohort effects follow second‐order random walk priors, allowing smooth temporal evolution while borrowing strength across adjacent intervals, and has been widely used for disease burden forecasting [24].

### 2.4 Statistical Analysis

Age‐standardized rates were expressed as the estimate per 100,000 populations and its 95% UI. All statistical analyses and data visualization were conducted using R software (version 4.4.0). The APC model analysis was performed using the Epi package, while data processing and visualization were carried out using the dplyr and ggplot2 packages.

## 3 Results

### 3.1 Global Burden of Brain and CNS Tumors

**Figure 1:**
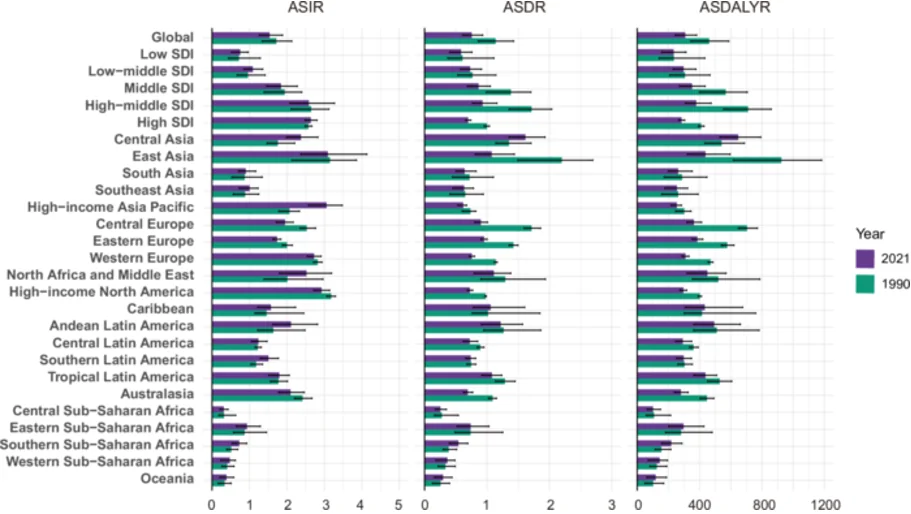
Global and regional age‐standardized rates (per 100,000) for CNS tumors. ASDALYR, age‐standardized DALY rate; ASDR, age‐standardized death rate; ASIR, age‐standardized incidence rate; CNS, central nervous system; DALY, disability‐adjusted life year; SDI, socio‐demographic index.

In 1990, an estimated 38816.64 (95% UI: 30302.58–48085.63) new cases of brain and CNS tumors occurred among children and adolescents worldwide. In contrast, the estimated new cases in 2021 were 40,534.53 (33,345.94–49,609.04). From 1990 to 2021, the age‐standardized incidence rate (ASIR) declined from 1.72 (1.34–2.13) to 1.54 (1.27–1.88) per 100,000, indicating a moderate global reduction in incidence. Over the same period, the age‐standardized death rate (ASDR) fell from 1.14 (0.86–1.43) to 0.76 (0.61–0.93), while the age‐standardized DALY rate (ASDALYR) decreased from 462.05 (344.49–586.83) to 308.37 (244.28–381.13) (Figure 1 and Table S1). These consistent declines suggest notable global progress in pediatric CNS tumor control.

To explore age‐specific patterns, all cases were divided into four age groups (0–4, 5–9, 10–14, and 15–19 years). Between 1990 and 2021, the ASIR in the 0–4‐year group declined from 2.47 (1.81–3.25) to 1.77 (1.36–2.25), and in the 5–9‐year group from 1.79 (1.39–2.22) to 1.68 (1.36–2.03). In contrast, mild increases were observed among adolescents aged 10–19 years (Table S2). The ASDR decreased by nearly 47% in the 0–4‐year group and by over 30% in the 5–9‐year group, but showed smaller declines in older age groups (Table S3). Similarly, the ASDALYR dropped by roughly 47% and 30% in the two youngest groups, whereas reductions were less than 15 per 100,000 in adolescents (Table S4).

### 3.2 Burden of Brain and CNS Tumors in Five SDI Quantiles and 21 GBD Regions

Across five SDI regions, the middle‐SDI regions consistently had the largest number of incident cases from 1990 to 2021. However, the ASIR displayed a positive gradient with development level—in 2021, high‐SDI regions recorded the highest ASIR (2.64 [2.49–3.80]), which was about 3.5 times higher than in low‐SDI regions (0.75 [0.52–0.96]) (Figure 1 and Table S1). Among age groups, the 0–4‐year group carried the heaviest burden, while the 15–19‐year group consistently showed the lowest ASIR. For instance, ASIRs ranged from 1.24 (0.80–1.69) to 2.90 (2.18–3.99) in the youngest group, compared with 0.41 (0.29–0.52) to 2.31 (2.13–2.47) in the oldest (Table S2).

Mortality patterns differed from incidence. In 2021, the high‐middle SDI regions reported the highest ASDR (0.93 [0.77–1.16]) (Figure 1 and Table S1). Over the past three decades, ASDRs among older children (> 10 years) increased slightly in low and low‐middle SDI regions, in contrast to declining trends observed elsewhere (Table S3). Overall, ASDALYRs declined by about 47% and 38% in high‐middle and middle SDI regions, respectively (Figure 1 and Table S1). Notably, DALYs remained most concentrated in the 5–9‐year group, while substantial reductions were observed among those aged 0–4 years (Table S4).

Across 21 GBD regions in 2021, ASIRs ranged widely—from 0.31 (0.21–0.43) to 3.09 (2.37–4.14)—with East Asia, High‐income Asia Pacific, and High‐income North America showing the highest rates. Central Europe showed one of the largest declines in ASIR from 1990 to 2021, whereas Central Asia exhibited a marked increase (Figure 1 and Table S1). Regarding mortality, Central Asia, Andean Latin America, North Africa, and the Middle East reported the highest ASDRs. These patterns should be interpreted with caution, as they may partly reflect variation in data completeness, case ascertainment, and health system capacity across regions. In contrast, East Asia and Central and Eastern Europe showed marked declines (Figure 1 and Table S1).

Patterns in ASDALYRs reflected these regional differences. The proportion of regions exceeding 400 DALYs declined from 52.4% in 1990% to 28.6% in 2021, indicating overall global improvement. However, Central Asia showed an increase of approximately 20%, whereas East Asia experienced a substantial reduction (52.7%) (Figure 1 and Table S1).

Across age groups, the burden of CNS tumors remained highest among younger children (< 10 years), particularly in the 5–9‐year group. For example, the median ASDR in this group was 0.89 (0.77–1.04), compared with less than 0.80 per 100,000 in the other three groups (Tables S2–S4).

### 3.3 National Burden of Brain and CNS Tumors

**Figure 2:**
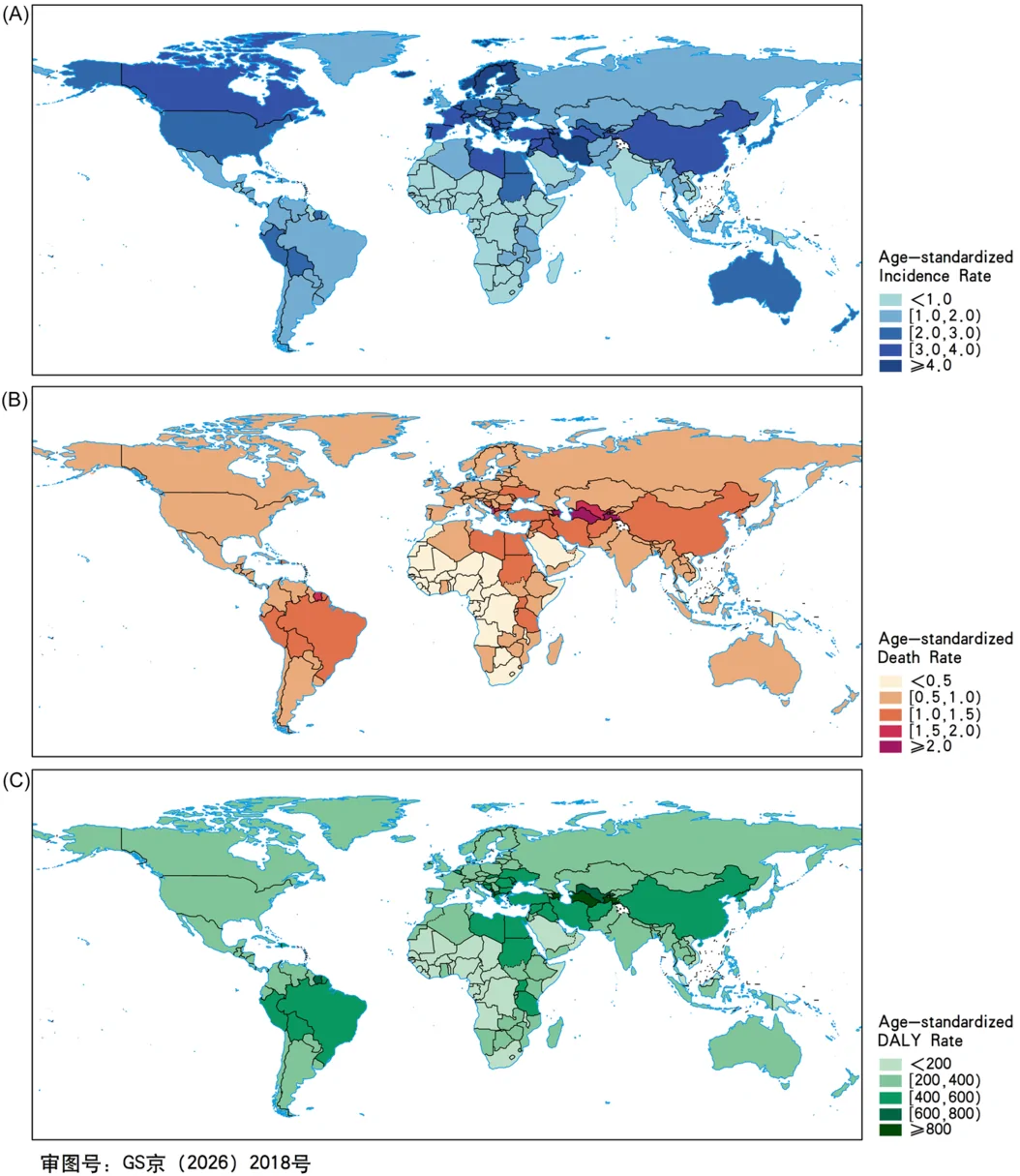
Global distribution of age‐standardized rates (per 100000) of CNS tumors in 2021. (A) Age‐standardized incidence rate in 2021; (B) Age‐standardized death rate in 2021; (C) age‐standardized DALY rate in 2021. ASDALYR, age‐standardized DALY rate; ASDR, age‐standardized death rate; ASIR, age‐standardized incidence rate; CNS, central nervous system; DALY, disability‐adjusted life year.

In 2021, the ASIR varied widely across countries. Among 204 countries and territories, 92 reported ≥ 50 new cases of CNS tumors (highest: China, India, and the United States), while 61 reported ≥ 50 deaths. From 1990 to 2021, the ASIR increased in 141 countries and territories, indicating a gradual global expansion of the disease burden. The number of high‐burden countries (ASIR > 3.00 per 100,000) rose from 27 in 1990 (peak: San Marino; Figure S1A) to 32 in 2021 (peak: Monaco, Norway, and San Marino; Figure 2A and Table S5).

Countries with large populations (> 50 million) contributed substantially to global incidence but tended to have moderate ASIRs. For instance, China's ASIR was 3.13 (95% UI: 2.39–4.21) in 2021, ranking 27th globally (Table S5). In 1990, 65 countries had an ASDR exceeding 1.00 per 100,000 (Figure S1B); by 2021, that number had decreased to 40 (Figure 2B). The highest ASDR in 2021 was observed in Tajikistan at 2.49 (95% UI: 1.73–3.69). Over the past 32 years, ASDRs increased in 78 countries, with the sharpest rises in Tokelau, Turkmenistan, and Niue, while the greatest reductions occurred in Greenland, Serbia, and China (Table S5).

The number of countries with an ASDALYR above 800 per 100,000 decreased from 11 in 1990 (Figure S1C) to five in 2021 (Figure 2C). The highest ASDALYRs in 2021 were found in Tajikistan, Tokelau, and Azerbaijan, whereas Serbia, Greenland, and China achieved one of the largest improvements, with China's ASDALYR decreasing by at least 53.19% (Table S5).

### 3.4 Global and Regional Trends in CNS Tumors Across Different Age Groups

Figure S2 and Table S6 summarize the temporal trends (net and local drifts) in incidence and mortality of CNS tumors across four age groups during 1992–2021. The APC analysis revealed heterogeneous patterns among SDI regions. Globally, the net drift of incidence was −0.13% per year (95% CI: −0.29% to 0.02%, *p* = 0.099), suggesting a mild but statistically nonsignificant decrease, with marked regional differences. Low and low‐middle SDI regions showed evident annual increases, while the middle and high‐middle SDI regions remained near stability (~0.10%). In local drift analysis, the steepest incidence decline occurred in the 0–4‐year group within low SDI regions (−0.76% [−0.98% to −0.53%] per year). Conversely, adolescents (15–19 years) showed a worrying global increase (0.48% [0.15% to 0.80%] per year), particularly in middle and high‐middle SDI regions (Figure S2A).

Mortality trends exhibited clearer improvement. The global net drift of mortality was −1.16% per year (95% CI: −1.35% to −0.98%, *p* < 0.0001). The decline was more pronounced in high‐middle SDI regions (−2.06% [−2.32% to −1.79%] per year). In contrast, low and low‐middle SDI regions showed small but concerning annual increases of 0.29% (0.11%–0.47%) and 0.21% (0.10%–0.33%), respectively.

For local drifts, the 0–4‐year group experienced the steepest mortality reduction globally (−1.86% [−2.14% to −1.59%] per year), corresponding to over 55% decrease in the last three decades. The high‐middle SDI region led this trend (−3.49% [−3.93% to −3.05%] per year). Alarmingly, the low SDI region was the only area showing a positive local drift in the 0–4‐year group, suggesting a continued deterioration of mortality burden in the world's least‐developed settings (Figure S2B).

### 3.5 Age, Period, and Birth Cohort Effects of Brain and CNS Tumors

**Figure 3:**
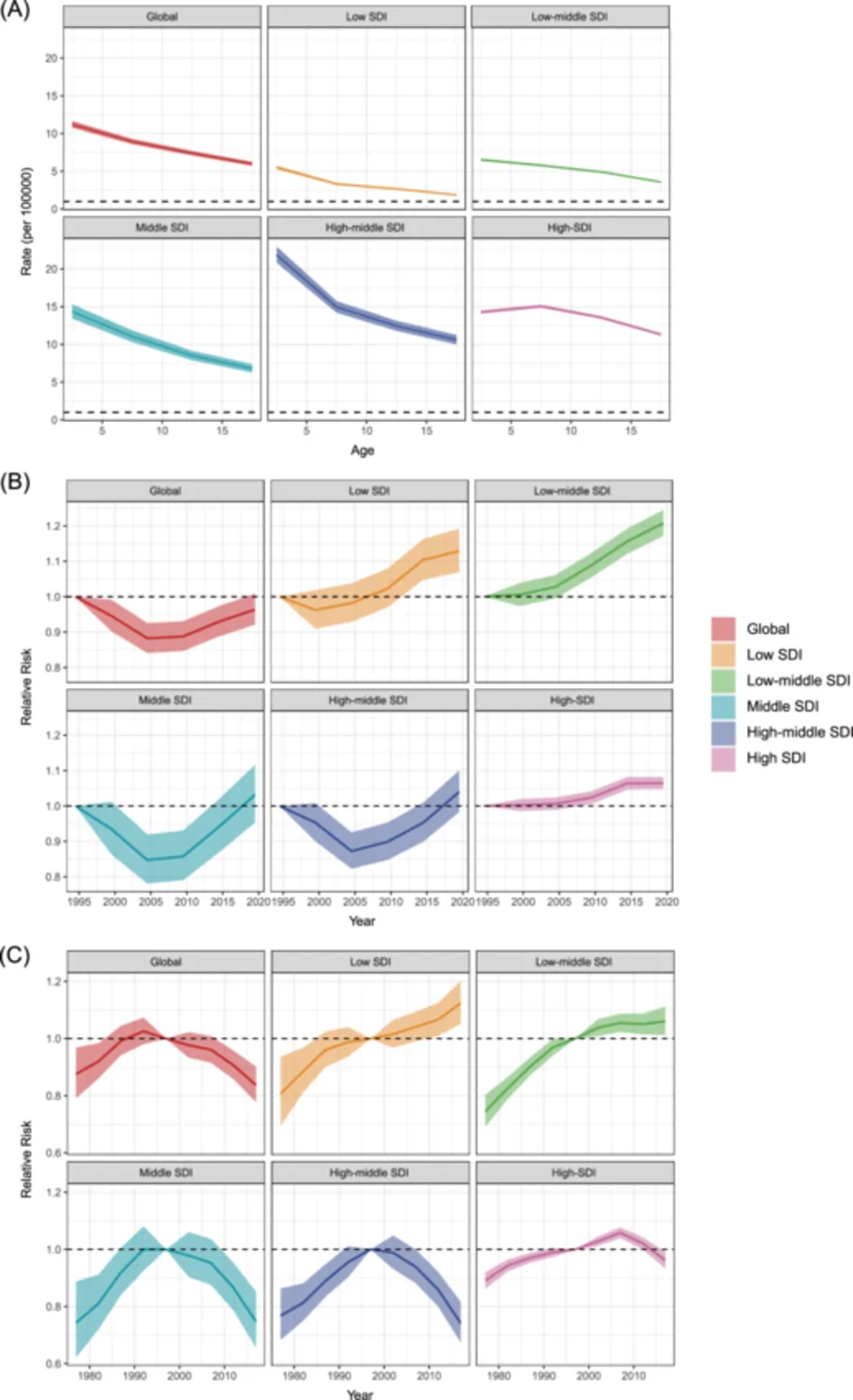
Age, period, and birth cohort effects on incidence of CNS tumors by SDI quintiles. (A) The age effect is depicted through the longitudinal rates specific to age, which are adjusted for variations across different birth cohorts, taking into account the period‐specific deviations. (B) Period effects are shown through the relative risk of mortality of brain and CNS tumors during different periods, calculated as the ratio of the age‐specific rates from the period from 1992 to 1996 to 2017–2021, with the baseline period set as 1992–1996. (C) Birth cohort effects are demonstrated by the cohort relative risk of mortality and calculated as the ratio of age‐specific rates from 1972 to 1981 cohort to 2012–2021 cohort, with the reference cohort set at 1992–2001. The dots and shaded areas denote the incidence rates or rate ratios and their corresponding 95% CIs. CNS, central nervous system; SDI, socio‐demographic index.

The age, period, and birth cohort effects on incidence are presented in Figure 3 and Table S9. Overall, similar age‐related patterns were observed across SDI quintiles, with the highest incidence risk in the 0–4‐year group, followed by a steady decline with increasing age. Notably, low‐SDI countries showed consistently lower incidence risks across all age groups compared to other regions (Figure 3A). Globally, period effects demonstrated a downward trend from 1992 to 2006, followed by a renewed increase from 2007 to 2021. Comparable fluctuations appeared in low, middle, and high‐middle SDI regions, while low‐middle and high SDI regions showed continuous increases, signaling potential challenges ahead. Compared with the reference period (1992–1996), the 2017–2021 period recorded the highest relative risks (RRs) in low‐middle (1.21, 95% CI: 1.17–1.25) and low SDI regions (1.13, 95% CI: 1.07–1.19), indicating less favorable epidemiologic trends (Figure 3B).

Birth cohort effects showed an inverted U‐shaped pattern globally and in middle and high‐middle SDI regions, while low and low‐middle SDI regions demonstrated a continuous rise. The highest RR was observed in the low‐SDI region (1.12, 95% CI: 1.05–1.20) among cohorts born in 2012–2021. Compared with the reference cohort (1992–2001), RRs for individuals born in 1997–2006, 2002–2011, and 2007–2016 were higher in the high‐SDI region (Figure 3C). These findings highlight widening disparities in pediatric CNS tumor incidence across socioeconomic strata.

The age effects on mortality largely paralleled those on incidence but exhibited steeper gradients. Generally, low‐SDI regions maintained the lowest mortality risks across all age groups (Figure S3A). However, mortality rates in middle and high‐middle SDI regions were higher for younger children (0–9 years) than in other SDI categories, particularly within the 0–4‐year group, suggesting poorer survival outcomes in early childhood. Despite an overall global decline in mortality, period effects varied across SDI levels. Compared with the reference period (1992–1996), RRs of mortality showed a gradual increase after an initial drop in low and low‐middle SDI regions (Figure S3B).

Cohort effects revealed significant mortality improvements worldwide, especially in middle, high‐middle, and high SDI regions. However, the low‐SDI region showed a mild yet persistent upward trend in RR (Figure S3C). These results indicate that, while global mortality from pediatric CNS tumors has declined across multiple dimensions, certain low‐resource regions remain vulnerable and require targeted public health interventions.

### 3.6 Prediction of Global ASIR, and ASDR up to 2036

**Figure 4:**
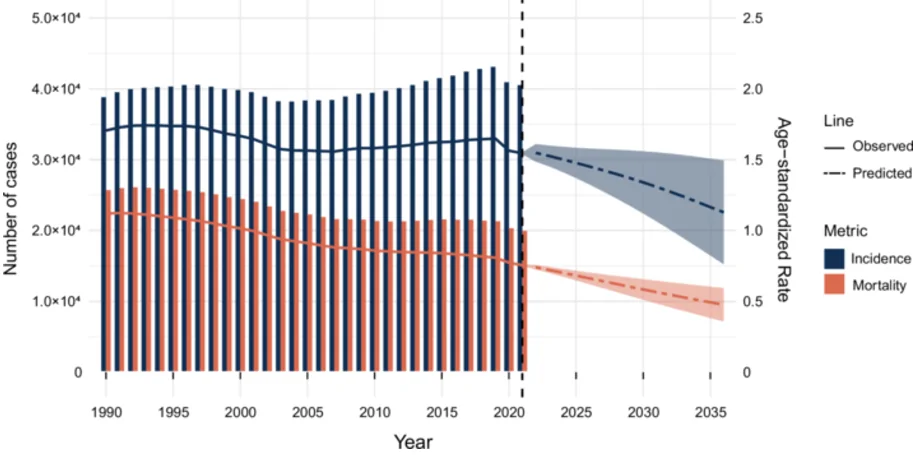
Projects the numbers and age‐standardized rates (per 100000) of CNS tumors from 2022 to 2036. CNS, central nervous system.

Using the BAPC model, we projected global trends of ASIR and ASDR for CNS tumors from 2022 to 2036 (Figure 4 and Table S7). The ASIR is expected to show a gradual reduction, reaching 1.13 (95% CI: 0.76–1.49) by 2036. In contrast, the decline in mortality is projected to be more pronounced, with the ASDR estimated at 0.48 (0.36–0.59)—representing a reduction of over one‐third compared to 2021. These projections suggest continued global improvement in pediatric CNS tumor outcomes, though progress may remain uneven across regions.

## 4 Discussion

In this study, we comprehensively described the global burden and epidemiological trends of CNS tumors among children and adolescents over the past 32 years. Overall, a global decline was observed, particularly in mortality and disability‐adjusted life years (DALYs). Regionally, high and high‐middle SDI areas demonstrated continuous improvement, whereas low and low‐middle SDI countries experienced an increasingly dire situation in recent years. Among all age groups, the youngest cohort (0–4 years) faced the highest overall risk but showed the most pronounced declines in both incidence and mortality. In contrast, older groups (10–19 years) exhibited either a mild increase in incidence or only modest reductions in mortality.

The higher incidence observed in high‐SDI regions should be interpreted with caution, as this pattern is critical to understanding global disparities. Differences in diagnostic intensity, including greater availability and utilization of neuroimaging, more comprehensive cancer registry coverage, and more standardized coding practices, are likely to contribute to higher case ascertainment in these settings. Consequently, incidence estimates may partly reflect differences in detection capacity rather than true underlying risk. In contrast, underdiagnosis and incomplete reporting in low‐SDI regions may lead to underestimation of the actual disease burden. These methodological considerations are therefore critical for properly contextualizing regional disparities and should be carefully accounted for when interpreting SDI‐related patterns.

At the country level, considerable variation was observed across SDI categories (Tables S9 and 10). Many high and high‐middle SDI countries, such as the United Kingdom and Iceland, as well as Italy and China, showed decreases in both ASIR and ASDR. Conversely, several countries—including Monaco, Japan, and Belarus—displayed increases in both ASIR and ASDR. These disparities mirror the persistent inequalities in economic development and healthcare resource distribution worldwide. In particular, when access to medical resources becomes a limiting factor, countries with higher population densities may experience greater pressure from mortality burdens.

Notably, patterns observed in children and adolescents may differ from those reported in all‐age populations [25]. Previous global assessments have suggested that, in the general population, the burden of CNS tumors is strongly influenced by aging and diagnostic capacity, whereas pediatric cases are more closely related to developmental and biological factors. While direct comparisons are limited by differences in age structure and disease composition, the relatively distinct trends observed in younger populations highlight the importance of age‐specific analyses. These differences underscore the need to interpret pediatric CNS tumor burden independently from all‐age estimates.

**Figure 5:**
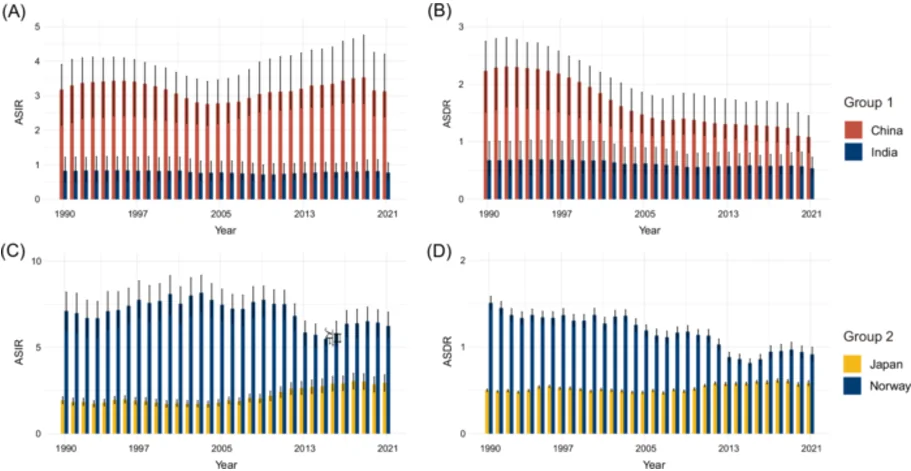
Comparison of ASIR and ASDR trends between different countries from 1990 to 2021. (A) ASIR of China and India from 1990 to 2021; (B) ASDR of China and India from 1990 to 2021; (C) ASIR of Japan and Norway from 1990 to 2021; (D) ASDR of Japan and Norway from 1990 to 2021. ASDR, age‐standardized death rate; ASIR, age‐standardized incidence rate.

To further examine differences in burden trends, two sets of countries were analyzed in greater detail. The first group, China and India, represents nations with large populations, while the second group, Japan and Norway, includes economically and medically advanced countries since the 1990s (Figure 5, Table S8). In China, ASIR displayed a cyclical pattern over roughly 15‐year intervals: an initial decline from 1990 to 2005, followed by an upward phase through the next 15 years, and an apparent decrease beginning around 2020. India showed a similar pattern, with a mild decline during the early 2000s and a gradual increase thereafter (Figure 5A). Both countries exhibited comparable ASDR trends. Since 1990, China's ASDR has dropped by more than half—from 2.23 (95% UI: 1.51–2.75) to 1.08 (0.81–1.45) in 2021—while India has achieved a steady 20% reduction over the same period (Figure 5B).

In Japan, ASIR decreased slightly for about 15 years before rising sharply to 2.95 (95% UI: 2.47–3.41) in 2021. ASDR remained relatively stable around 0.50 for nearly two decades but increased by nearly 20% to 0.58 (0.55–0.61) by 2021. In contrast, Norway showed a fluctuating yet overall declining trend in both morbidity and mortality, suggesting progressive improvements in disease burden (Figure 5C,D).

Among the selected countries, both China and India showed rising ASIR and ASDR, likely linked to their rapid economic growth, substantial improvements in healthcare systems, advancements in medical research, and expanding international collaboration. In contrast, despite Japan's well‐developed economy and healthcare infrastructure, both ASIR and ASDR have worsened. Several hypotheses may help to explain these trends, including changes in diagnostic practices and increased detection associated with wider availability of neuroimaging, as well as potential shifts in environmental exposures or population‐level risk factors. In addition, improvements in case ascertainment and reporting completeness over time may also contribute to the observed increases. These factors warrant further investigation to better understand the drivers of disease burden in this setting.

The apparent increase in CNS tumor detection in low and low‐middle SDI countries may partly reflect better diagnostic capabilities, which does not necessarily reflect a true increase in disease burden. However, the rising mortality burden in recent decades is of greater concern. Globally, the increased ASIR observed in low, low‐middle, and high SDI countries might be associated with environmental factors, such as excessive early exposure to electronic devices, environmentally induced genetic alterations, pollution, and ionizing radiation [15, 26]. The countertrend observed between ASDR and ASIR may largely reflect improved access to primary health care, enhanced medical coverage, and progress in surgical techniques, high‐quality nursing [27], and multidisciplinary treatment. For instance, the wide application of imaging technology not only enhances the diagnostic rate but also makes early intervention feasible.

In countries with persistently high or rising mortality, priorities should include advancing surgical techniques, adopting standardized and effective treatment protocols, improving postoperative management, preventing complications, and increasing medical investment—especially in resource‐limited regions. These measures are crucial to mitigating mortality.

Driven by improved medical standards, major advances in basic research, extensive drug development, clinical trials, and continuous progress in surgery and postoperative care, numerous breakthroughs in CNS tumor management have markedly prolonged survival among affected patients [28, 29, 30, 31, 32]. Moreover, emerging technologies and discoveries are transforming the understanding of CNS tumors and providing a foundation for more effective therapies [33, 34, 35, 36].

Looking ahead, efforts should focus on strengthening health systems rather than pursuing population‐level screening strategies unsupported by current evidence. Improving diagnostic capacity, streamlining referral pathways, and expanding access to specialized pediatric neuro‐oncology services remain central to achieving timely diagnosis of CNS tumors in children. In economically underdeveloped regions, greater investment in radiotherapy infrastructure, multidisciplinary care, and supportive services is particularly needed to address persistent disparities in outcomes. In addition, previous studies have also suggested possible associations between CNS tumor incidence and factors such as electronic device use, radiofrequency electromagnetic radiation, and environmental exposures [26, 37]. Although these cannot be interpreted as causal based on Global Burden of Disease analyses, they may be more appropriately viewed as hypotheses that merit further investigation to clarify the underlying etiological mechanisms.

In light of these findings, while continued exploration of CNS tumor etiology remains essential, future health policies should prioritize reducing mortality and minimizing fatal or disabling treatment complications. Ongoing industrialization, particulate pollution, and electromagnetic exposure may further increase the incidence of CNS tumors, particularly in low and low‐middle SDI regions [38]. Addressing both morbidity and mortality simultaneously is key to effectively lowering the global burden of CNS tumors and advancing progress toward the 2030 Sustainable Development Goals [39].

There are several limitations in the study. The analysis described the burden and temporal trends of CNS tumors but did not address etiology or environmental risk factors, as the Global Burden of Disease framework does not yet attribute these to brain tumors in a standardized way. The underlying data also carry inherent uncertainty. Because the estimates draw on heterogeneous sources, differences in extraction methods and in the reliability of the original studies are unavoidable; these are compounded by the different diagnostic and reporting standards adopted across countries and institutions, so that the reported figures may vary considerably across regions. A portion of the observed variation in incidence and mortality is likely to arise from changes in diagnostic and screening infrastructure, particularly the broader availability of neuroimaging, rather than from genuine shifts in disease risk; this concern is most salient where diagnostic capacity has expanded rapidly.

## 5 Conclusions

In summary, children under 10 years of age show notably higher age‐standardized incidence and death rates of CNS tumors, and the increasing burden in countries with lower sociodemographic index levels calls for focused attention on these young and vulnerable populations. Although global ASIR and ASDR are projected to decline by 2036, the disparities across regions remain concerning. These findings highlight the need for targeted public health strategies, equitable resource allocation, and strengthened international cooperation to reduce disease burden and improve cancer care outcomes.

## Author Contributions

**Zhaoyang Feng:** conceptualization, data curation, methodology, software, visualization, writing – original draft. **Yu Su:** conceptualization, data curation, methodology, visualization, writing – review and editing. **Lin Yang:** methodology, supervision. **Kaiwen Deng:** formal analysis, writing – review and editing. **Jianmin Wang** and **Dongyang Wang:** supervision. **Jiao Zhang:** resources, software. **Fei Liu:** investigation, supervision. **Yuyan Liang:** investigation. **Wei Wang:** funding acquisition, writing – review and editing. **Xiaoguang Qiu:** investigation, writing – review and editing. **Tao Jiang** and **Hailong Liu:** funding acquisition, project administration, supervision, writing – review and editing. **Yu Tian:** funding acquisition, methodology, project administration, supervision, writing – review and editing.

## Ethics Statement

The authors have nothing to report.

## Consent

The authors have nothing to report.

## Conflicts of Interest

The authors declare no conflicts of interest.

## Supporting information


**Figure S1:** Global distribution of age‐standardized rates (per 100,000) of CNS tumors in 1990. (A) Age‐standardized incidence rate in 2021. (B) Age‐standardized death rate in 2021. (C) Age‐standardized DALY rate in 2021. ASDALYR, age‐standardized DALY rate; ASDR, age‐standardized death rate; ASIR, age‐standardized incidence rate; CNS, central nervous system; DALY, disability‐adjusted life year.

**Figure S2:** Local drift and age distribution for CNS tumors from 1992 to 2021 across SDI quintiles. (A) Local drift of incidence from 1992 to 2021 for CNS tumors. (B) Local drift of mortality from 1992 to 2021 for CNS tumors. The dots and shaded areas indicate the annual percentage change (% per year) and the corresponding 95% CIs. CNS: central nervous system; SDI: socio‐demographic index.

**Figure S3:** Age, period, and birth cohort effects on morality of CNS tumors by SDI quintiles. (A) The age effect is depicted through the longitudinal rates specific to age, which are adjusted for variations across different birth cohorts, taking into account the period‐specific deviations. (B) Period effects are shown through the relative risk of mortality of brain and CNS tumors during different periods, calculated as the ratio of the age‐specific rates from the period from 1992–1996 to 2017–2021, with the baseline period set as 1992–1996. (C) Birth cohort effects are demonstrated by the cohort relative risk of mortality and calculated as the ratio of age‐specific rates from 1972–1981 cohort to 2012–2021 cohort, with the reference cohort set at 1992–2001. The dots and shaded areas denote the incidence rates or rate ratios and their corresponding 95% CIs. CNS, central nervous system; SDI, socio‐demographic index.

**Table S1:** The numbers and age‐standardized rates with 95% UI for CNS tumors in 1990 and 2021. Abbreviations: ASDALYR, age‐standardized DALY rate; ASDR, age‐standardized death rate; ASIR, age‐standardized incidence rate; CNS, central nervous system; DALY, disability‐adjusted life year; UI, uncertainty interval.

**Table S2:** Age‐standardized incidence rate of CNS tumors across four age groups in 1990 and 2021. Abbreviation: CNS, central nervous system.

**Table S3:** Age‐standardized death rate of CNS tumors across four age groups in 1990 and 2021. Abbreviation: CNS, central nervous system.

**Table S4:** Age‐standardized DALY rate of CNS tumors across four age groups in 1990 and 2021. Abbreviations: CNS, central nervous system; DALY, disability‐adjusted life year.

**Table S5:** Age‐standardized rates of CNS tumors of age < 20 years in 1990 and 2021. Abbreviations: ASDALYR, age‐standardized DALY rate; ASDR, age‐standardized death rate; ASIR, age‐standardized incidence rate; CNS, central nervous system; DALY, Disability‐adjusted life years.

**Table S6:** Net drifts and local drifts of CNS tumors from 1992 to 2021 across SDI quintiles. The value of the net drift and local drift indicates the average annual percentage change (% per year), and the corresponding 95% CIs are displayed by “CI_Low” and “CI_High.” Abbreviations: CI, confidence interval; CNS, central nervous system; SDI, socio‐demographic index.

**Table S7:** Observed and projected (2022–2036) global age‐standardized rates of CNS tumors from 1990 to 2036. Abbreviation: CNS, central nervous system.

**Table S8:** ASIR and ASDR of CNS tumors between different countries from 1990 to 2021. Abbreviations: ASDR, age‐standardized death rate; ASIR, age‐standardized incidence rate; CNS, central nervous system.

**Table S9:** The numbers of age, period, and birth cohort effects of CNS tumors by SDI quintiles. The “Rate” and “RateRatio” indicate the estimated value by age‐period‐cohort model, and the corresponding 95% CIs are displayed by “CI_Low” and “CI_High.” Abbreviations: CI, confidence interval; CNS, central nervous system; SDI, socio‐demographic index.

**Table S10:** SDI values for all estimated GBD 2021 locations and the range of different SDI quintiles. Abbreviation: SDI, socio‐demographic index.

## Data Availability

Data on brain and CNS tumors from 1990 to 2021 period were obtained through the Global Health Data Exchange (GHDx) query tool (https://vizhub.healthdata.org/gbd-results/) [17], including annual statistics on incidence, mortality, and DALYs. Other data, including age‐standardized rates, results from age‐period‐cohort model, and projections are performed by authors, and uploaded and available as Supporting materials.
