# Supplementary material for: Global Burden and Temporal Trends of Brain and Central Nervous System Tumors in Children and Adolescents: A Systematic Analysis for the Global Burden of Disease Study 2021
Source: Cancer Innov. 2026 Aug 23;5(4):e70079. doi: 10.1002/cai2.70079 (PMC13499956; doi:10.1002/cai2.70079)
Supplement: Supplementary file 1 [file CAI2-5-e70079-s003.pdf]

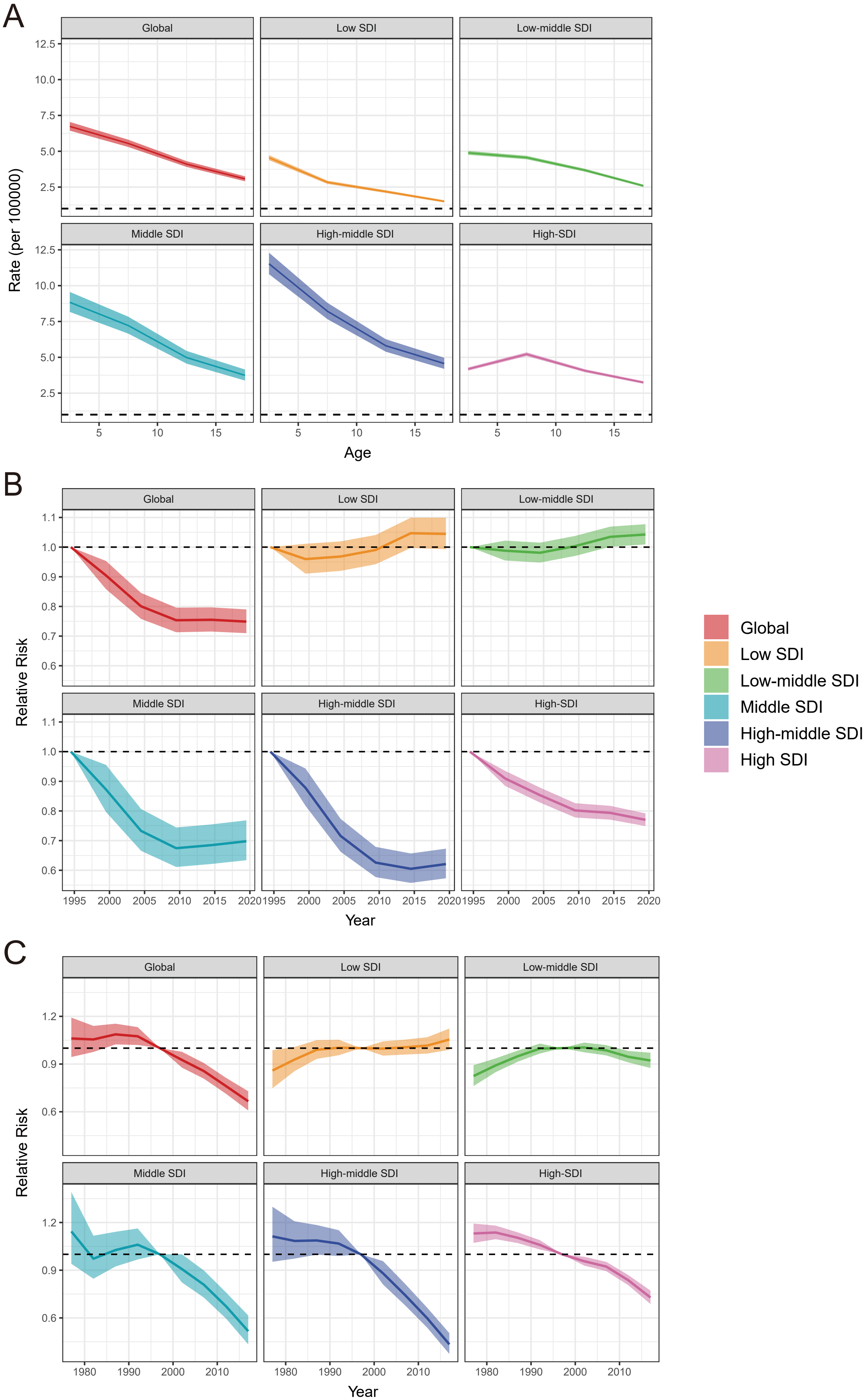
A
Global
Low SDI
Low-middle SDI
Middle SDI
High-middle SDI
High-SDI
Rate (per 100000)
12.5
10.0
7.5
5.0
2.5
5
10
15
Age
B
Relative Risk
1.1
1.0
0.9
0.8
0.7
0.6
1995
2000
2005
2010
2015
2020
Year
C
Relative Risk
1.2
0.9
0.6
1980
1990
2000
2010
Year
Global
Low SDI
Low-middle SDI
Middle SDI
High-middle SDI
High SDI
