# Supplementary material for: Global Burden and Temporal Trends of Brain and Central Nervous System Tumors in Children and Adolescents: A Systematic Analysis for the Global Burden of Disease Study 2021
Source: Cancer Innov. 2026 Aug 23;5(4):e70079. doi: 10.1002/cai2.70079 (PMC13499956; doi:10.1002/cai2.70079)
Supplement: Supplementary file 2 [file CAI2-5-e70079-s005.pdf]

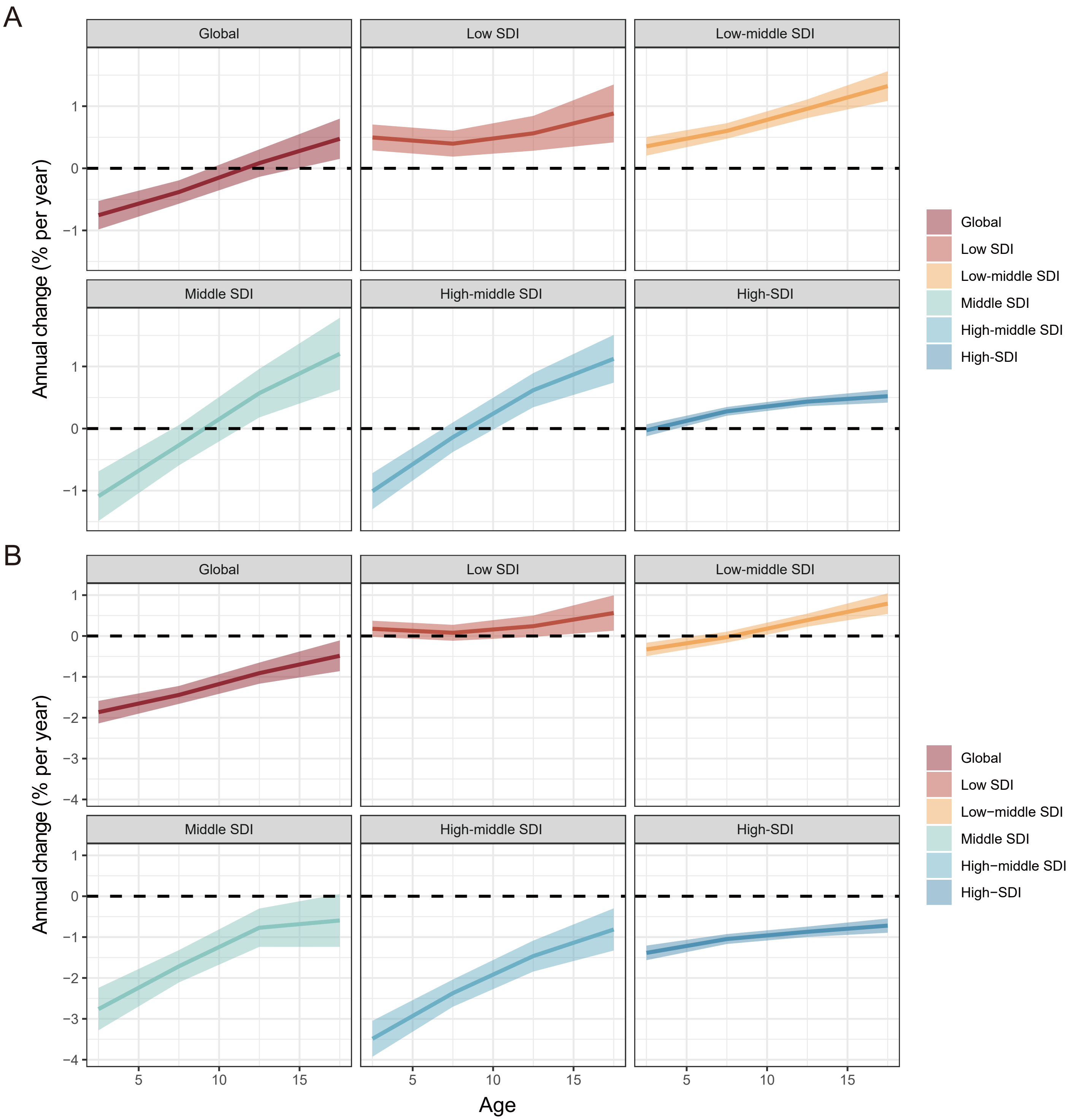
A
Global
Low SDI
Low-middle SDI
Middle SDI
High-middle SDI
High-SDI
Annual change (% per year)
1
0
−1
Global
Low SDI
Low-middle SDI
Middle SDI
High-middle SDI
High-SDI
B
Global
Low SDI
Low-middle SDI
Middle SDI
High-middle SDI
High-SDI
Annual change (% per year)
1
0
−1
−2
−3
−4
5
10
15
Age
Global
Low SDI
Low−middle SDI
Middle SDI
High−middle SDI
High−SDI
